# *Lotus* Base: An integrated information portal for the model legume *Lotus japonicus*

**DOI:** 10.1038/srep39447

**Published:** 2016-12-23

**Authors:** Terry Mun, Asger Bachmann, Vikas Gupta, Jens Stougaard, Stig U. Andersen

**Affiliations:** 1 Department of Molecular Biology and Genetics, Aarhus University, Gustav Wieds Vej 10, DK-8000 Aarhus C, Denmark; 2 Bioinformatics Research Centre, Aarhus University, C. F. Møllers Allé 8, DK-8000 Aarhus C, Denmark

## Abstract

*Lotus japonicus* is a well-characterized model legume widely used in the study of plant-microbe interactions. However, datasets from various *Lotus* studies are poorly integrated and lack interoperability. We recognize the need for a comprehensive repository that allows comprehensive and dynamic exploration of *Lotus* genomic and transcriptomic data. Equally important are user-friendly in-browser tools designed for data visualization and interpretation. Here, we present *Lotus* Base, which opens to the research community a large, established *LORE1* insertion mutant population containing an excess of 120,000 lines, and serves the end-user tightly integrated data from *Lotus*, such as the reference genome, annotated proteins, and expression profiling data. We report the integration of expression data from the *L. japonicus* gene expression atlas project, and the development of tools to cluster and export such data, allowing users to construct, visualize, and annotate co-expression gene networks. *Lotus* Base takes advantage of modern advances in browser technology to deliver powerful data interpretation for biologists. Its modular construction and publicly available application programming interface enable developers to tap into the wealth of integrated *Lotus* data. *Lotus* Base is freely accessible at: https://lotus.au.dk.

*Lotus japonicus* is a popular, well-characterized model legume^1^, widely used to study plant-microbe interactions due to its ability to establish a range of different types of relationship with microorganisms along the symbiosis–pathogenesis spectrum—ranging from biological nitrogen fixation^2^ and arbuscular mycorrhizal symbiosis^3^, to bacterial^4^ and fungal^5^ pathogenesis. The establishment of the *LORE1* mutant population^6^,^7^,^8^ and the annotated sequence of the *Lotus japonicus* genome^9^ necessitated a centralized and freely available online resource for researchers working with this model legume. From its original incarnation as a *LORE1* resource site to allow handling and processing of *LORE1* mutant seeds orders, *Lotus* Base has grown to incorporate additional resources and toolkits tailored for the general needs of the research community. Although various *Lotus* databases have been made available to the public through different providers—such as the v3.0 genome through the Kazusa DNA Research Institute^9^; and the *L. japonicus* Gene Expression Atlas^10^, there is hitherto no publicly accessible repository to integrate all these data in a coherent manner. Due to the lack of a central information portal for *Lotus japonicus*—in spite of its popularity and utility as a model plant organism^11^,^12^,^13^, and its role in the study of biological nitrogen fixation^14^—we believe that *Lotus* Base is poised to benefit a large research community that does not traditionally have convenient access to such data.

*Lotus* Base is designed to be a user-friendly browser-based application that is operating system (OS)-agnostic and publicly accessible. In order to improve the workflow of researchers, *Lotus* Base provides functionalities that enable users to (1) search and retrieve sequence information; (2) identify functions and co-expression of *Lotus* gene(s) of interest; (3) view and order *LORE1* lines that contain insertions in candidate gene(s); (4) visualize and annotate co-expression networks in *Lotus*; and (5) view and investigate gene structures and annotations of the latest *Lotus* genome version. In order to present a unified workflow, *Lotus* Base is designed with deep linking in mind where various toolkits can exchange information with each other. The secure and ethical design behind *Lotus* Base ensures that user information and credentials are properly stored and cryptographically encrypted during transmission, and that users have free access to, and retain ownership of, the data they have generated.

As a wide spectrum of technological competencies exist across the board within the research community, compounded by differential access to various computing technologies among researchers, *Lotus* Base was built from ground up with a focus on making tools simple to use, yet sufficiently verbose, for a common end-user. In short, *Lotus* Base ensures secure yet convenient access to *Lotus* genomics and expression data made coherent by deep linking by leveraging the latest browser technologies, avoiding the need for tedious software updates for related dependencies and/or plugins.

## Methods & Data

### Technologies

*Lotus* Base adopts a clean, minimal design principle for the front-end design, allowing users who are accustomed to normal browser use to acquaint themselves with the resource easily. *Lotus* Base is designed to be used by modern standards-compliant browsers, and is powered by Apache running on a CentOS7 server behind a load balancer. All communications between the end-user and our front-facing load balancer are SSL encrypted, while the load balancer communicates with our web server by normal HTTP protocols. Our database adopts an atomic design and is powered by either MySQL or PostgreSQL, depending on the needs of individual applications. In addition, a Python and R stack known as Anaconda^15^, powers some *Lotus* Base functionalities.

On the client end, we are serving pages via PHP 5.6, using HTML5 and CSS3, with user interactions assisted and enhanced with asynchronous JavaScript and XML (AJAX) and jQuery. We have implemented HMAC-SHA256 encryption^16^ for generation and verification of RFC 7519-compliant JSON web tokens (JWT)^17^ for user and API key authentication. Server-based sessions are frequently cycled to avoid session hijacking. All user login credentials are individually salted and cryptographically hashed, and are never stored or transmitted in plain text format.

*Lotus* Base is built using Grunt^18^, while the developer blog and application programming interface (API) documentation are generated by Jekyll^19^ and Slate^20^ respectively. Source control is done via git. The resource is designed to be extensible and modular, with the code base made open source through a GitHub repository (https://github.com/lotusbase/lotus.au.dk). Other features of *Lotus* Base are powered by various open-source projects, which together bring about a friendly, dynamic, and coherent user experience.

### Overview of data provisioned by *Lotus* Base

**Figure 1:**
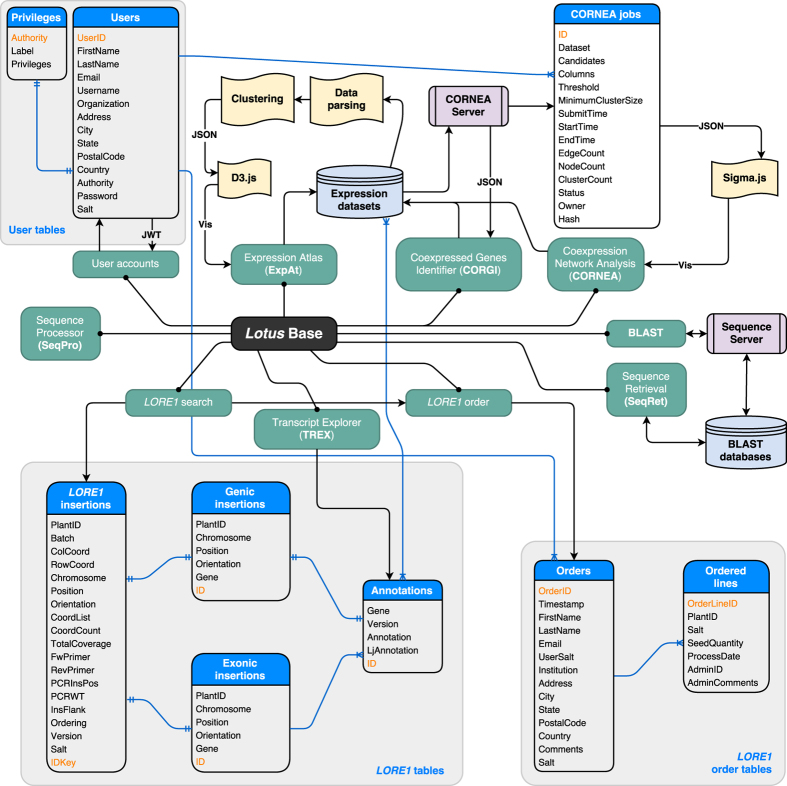
The server-side design behind *Lotus* Base. The resource consists of several tools deeply integrated with each other—*LORE1* search, *LORE1* order, Sequence Retrieval (SeqRet), BLAST, CORx toolkit (CORGI and CORNEA) and Expression Atlas (ExpAt). MySQL tables are indicated in blue entity boxes with column names listed. Highlighted column names, in orange, are used as primary indexes. Tables are grouped by the function they serve, in relation to individual tools. Due to space restraints, expression datasets are described in further detail in Fig. 4. An overview of all integrated datasets on *Lotus* Base is available in Table 1.

In the backend, *Lotus* Base constitutes various deeply integrated toolkits, which provide a coherent and simple workflow (Fig. 1). *Lotus* data are made publicly available (v2.5 of *L. japonicus* genome and proteins; and v3.0 of the following *L. japonicus* databases: genome, proteins, cDNA, and coding sequences). All available *Lotus* data that have been integrated to *Lotus* Base are outlined in Table 1.

### Genomic data

*Lotus* Base currently hosts the two latest versions of the *L. japonicus* genome assembly, versions 2.5 and 3.0 respectively. Both versions of the genome comprise six chromosomes and a single artificial chromosome 0 containing unassembled contigs interspersed with N spacers, with version 3.0 containing an additional mitochondrion genome. Version 2.5 of the genome includes sequence information from transformation-competent/bacterial artificial chromosome (TAC/BAC) clone Sanger-sequencing data, amounting to a total genome size of 397 Mb. Meanwhile, version 3.0 was assembled by integrating sequencing data from both TAC/BAC clone Sanger-sequencing and Illumina shotgun sequencing of up to 40× coverage, amounting to a total genome size of 448 Mb.

### Genes and predicted proteins

Gene features such as mRNA, alternatively spliced transcripts (also known as isoforms), exons, and coding sequences were made available both in the form of (1) a GFF3 file, used in a customized JBrowse^21^ implementation; and (2) individual BLAST databases. Gene and protein predictions were based on Augustus^22^, Cufflinks^23^, Genemark^24^, and Glimmer^25^. *Lotus* Base currently hosts gene and protein predictions for two versions of the genome assembly—19,713 predicted genes and 38,482 transcripts for v2.5; 44,483 predicted genes and 98,302 transcripts for v3.0.

### *LORE1* resource

*Lotus* Base is an integrated, one-stop platform for the *LORE1* insertional mutagenesis population, hitherto the largest plant mutagenesis population established (Table 2). The *LORE1* insertional mutagenesis population and its accompanying data (Table 3), collectively known as the *LORE1* resource, have been previously described^8^. *Lotus* Base hosts 121,531 mutant lines containing 629,631 unique insertions, sourced from 14 Danish (DK01–03, 05, 07–16; 108,133 lines) and 3 Japanese (JPA, JPL, and JPP; 13,398 lines) batches^26^. All *LORE1* lines have been sequenced and the ± 1000 bp flanking sequences were used for automated primer design using Primer3^27^. In addition, all *LORE1* associated data can be downloaded at https://lotus.au.dk/data/lore1. The *LORE1* resource on *Lotus* Base has so far delivered more than 185,000 seeds from 3,800 unique mutant lines, shipped to 21 countries worldwide. The resource has also seen its use in several reverse genetics studies^28^,^29^,^30^,^31^,^32^.

**Figure 2:**
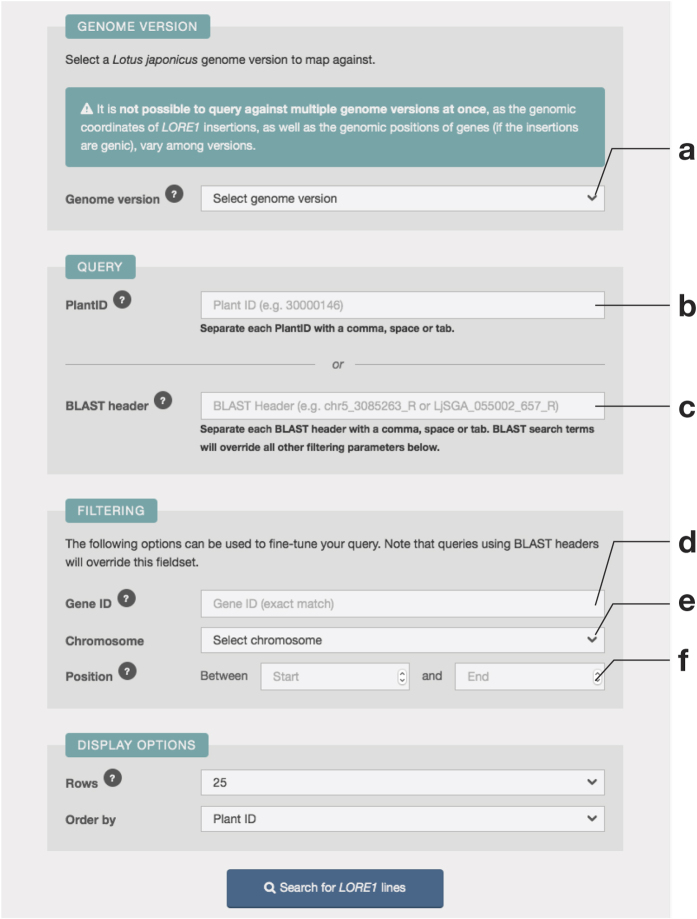
The *LORE1* search form. Field A is compulsory, while fields B and C are mutually exclusive. Fields D, E and F are optional fields that allows user to further filter their results if desired. (**a**) A dropdown menu for the *L. japonicus* genome version—currently v2.5 and v3.0 are publicly available. (**b**) The unique eight-digit identifier of a *LORE1* mutant line. One mutant line may have multiple BLAST headers. (**c**) A BLAST header, which is a unique *LORE1* insertion identifier, is an underscore-delimited string of chromosome, position and orientation of the insert. Each BLAST header should uniquely map back to a single *LORE1* insertion. (**d**) Filtering for *LORE1* inserts that are inserted in a gene of interest. The gene identifier differs among *L. japonicus* genome versions. (**e**) The chromosome where the *LORE1* insert is located in. (**f**) The genomic interval (inclusive on both ends), where the *LORE1* insert must be located in. If only one value is provided (be it in the “start” or “end” field), then a specific genomic coordinate is enforced.

With such a large volume of data available, the *LORE1* search form is designed to be intuitive and easy to use, allowing users to search for *LORE1* lines of interest using a variety of user-defined criteria (Fig. 2). Users may search for *LORE1* insertions based on: (1) a *LORE1* mutant line identifier; (2) an insertion identifier, also known as a BLAST header, which is an underscore delimited string containing the chromosome, position and orientation of a *LORE1* insert); (3) or the gene(s), if any, that the insertion is located in. Due to spatial constraints, data from all fields are not displayed on the search results page, although data export options are available on all pages.

Orders can be placed on all Danish *LORE1* lines (108,133; 89% of listed lines) for which seed stocks are available. Listed Japanese lines (13,398; 11% of listed lines) are included in our database but are not available for ordering—users are instead directed to LegumeBase (https://www.legumebase.brc.miyazaki-u.ac.jp/lore1BrowseAction.do) for ordering said lines.

### Expression data

*Lotus* Base also offers *Lotus*-related expression data sourced from various studies. The first dataset was derived from the *L. japonicus* gene expression atlas (LjGEA) project^10^, which combined expression data from additional studies^33^,^34^,^35^,^36^,^37^. The whole LjGEA dataset consists of 81 conditions sourced from 6 independent published studies^10^, such as the investigation of draught responses^33^, effect of mycorrhizal and symbionts inoculation^34^,^35^, transcriptome changes in symbiosis defective mutants^35^, effect of salt and nitrate treatment^35^,^36^,^37^, and transcriptome regulation in various plant organs^10^. We have mapped probe identifiers from the LjGEA dataset against the annotated proteins of *L. japonicus* genome v3.0 by performing BLAST alignments of LjGEA probe set against the predicted transcripts from *L. japonicus* genome v3.0 and selecting for hits with the lowest E-value(s). In addition to the LjGEA dataset, we have also integrated expression data from *Lotus* roots in response to germinating spore exudates from arbuscular mycorrhiza^5^, containing 3 conditions.

### Genome browser

The *Lotus* genome browser is powered by a customized version of JBrowse v1.12.0^21^, with the following tracks publicly available: *L. japonicus* MG20 reference genome v3.0; predicted protein tracks; *LORE1* insertions; genome gaps; repeat masks; and *L. japonicus* Gifu and MG20 RNAseq reads.

### *Lotus* BLAST and SeqRet, an improved NCBI BLAST and sequence retrieval tool

SequenceServer v1.0.4^38^ was modified according to our needs and serves as the backbone for *Lotus* Base Basic Local Alignment Search Tool (BLAST). *Lotus* BLAST currently runs using NCBI BLAST+ v2.2.31 executables^39^, allowing users to execute the total suite of BLAST algorithms—blastn, blastx, tblastn, tblastx, and blastp. Various toolkits on our site are integrated with an in-house developed Sequence Retrieval (SeqRet) tool, which allows real time retrieval of accession/identifier-based sequence information across all locally hosted *Lotus* BLAST databases. Users are presented with the option to view retrieved sequences in a modal box, or to download them as FASTA files for storage and/or further processing.

### Sequence Processor (SeqPro)

The traditional wwwblast package from NCBI still outputs BLAST results in a monospaced, plain text format that can be problematic to parse for the end user. Users carrying such data from other sites may encounter difficulty in extracting useful sequence identifiers. Sequence Processor (SeqPro) tool is designed as a regular-expression based parser to handle wwwblast output and provide a tabular output. In addition, SeqPro also helps to remove line breaks and number lines from plain text FASTA outputs, which improved readability of sequences if users simply want to store the nucleotide/amino acid sequences without any accompanying metadata such as row counts, nucleotide position numbers, and unnecessary line breaks.

### Transcript Mapper (TRAM)

As each *Lotus* genome assembly comes with a unique combination of predicted gene/transcript nomenclature and populations, we have designed a simple tool to aid users in mapping v2.5 to v3.0 transcripts and vice versa. A mapping table has a many-to-many relationship is precomputed by performing BLAST alignments between transcripts from both versions, and storing the highest confidence hits for all transcripts.

### Transcript Explorer (TREX)

For users to glean quick information about their genes or transcripts of interest, we have designed the Transcript Explorer (TREX) tool, which is simply a full-text search engine that allows users to pull integrated information related to their search candidates. The search result is tabulated and summarized to display the working name (if any), and the function of the candidate gene/transcript, its position in the *Lotus* genome and any *LORE1* lines with exonic insertions in the gene. Further information and deep links to other toolkits on the site, such as to ExpAt, *LORE1* search, individual gene pages, are available in a dropdown menu for each candidate.

### Expression Atlas (ExpAt)

We have developed a data-driven, web-based visualization tool for *L. japonicus* expression data. Visualization in the *L. japonicus* Expression Atlas (ExpAt) tool is powered by jQuery and d3.js^40^. The use of client-side JavaScript enables intuitive and dynamic customization, on-the-fly asynchronous clustering, and vector graphics export options—all of which are features unavailable in currently available expression data visualization tools for *Lotus*.

### Search functionalities

**Figure 3:**
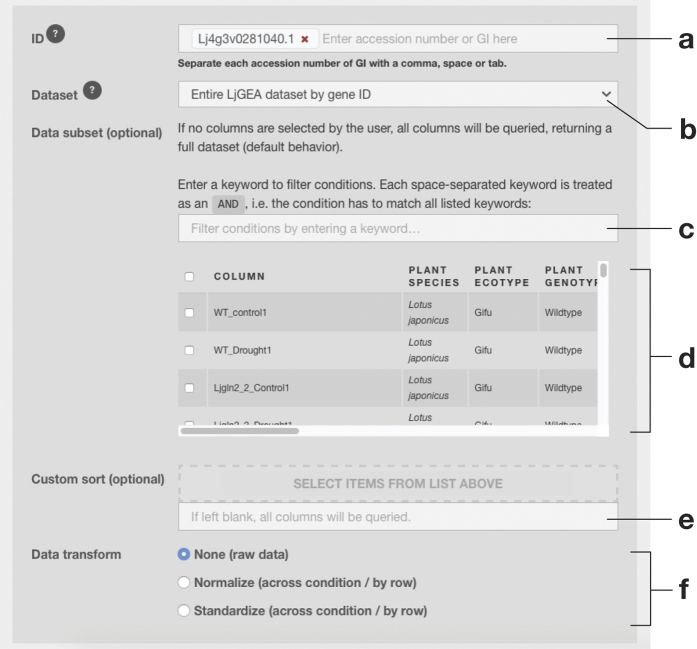
The design of ExpAt search form. (**a**) The query for the expression level of candidate(s) of interest—gene, transcript, or probe identifiers are accepted. (**b**) A dropdown selection menu for an ExpAt dataset to base the query upon. When a dataset is selected, the metadata table in (d) will be asynchronously updated with the related metadata from the selected dataset. (**c**) A text field to perform full-text search, using user defined keywords, in order to filter the columns in the metadata table. (**d**) The metadata table containing column/condition-associated data. (**e**) A text field that accepts a comma-separated string of columns generated from a previous ExpAt search, if a certain sorting order of columns is desired. Users may also drag to reorder checked columns from the metadata table. (**f**) An option to transform the expression levels.

ExpAt features a simple search form to query the expression levels of candidates (genes or probes, depending on the dataset selected) against a list of published datasets (Fig. 3). The user can subset a dataset by checking individual conditions, which can also be filtered by user-defined keyword(s) using an in-browser full-text search engine implemented using Lunr.js^41^.

### Table design

**Figure 4:**
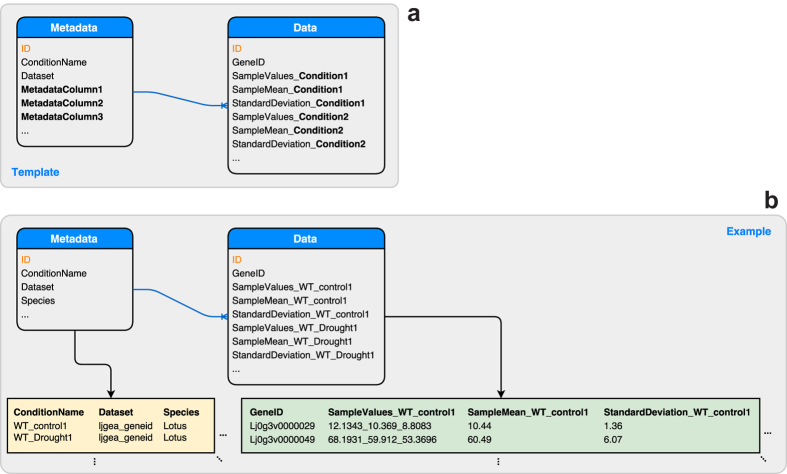
The organization of multi-dimensional expression data in the Expression Atlas (ExpAt) tool. A two-table system is used—the “metadata” table is used to store metadata associated with each condition. The “data” table is used to store expression levels associated with each row identifier. Highlighted column names, in orange, are used as primary indexes. (**a**) A standard template used for all ExpAt datasets. (**b**) An example of what an ExpAt dataset may look like, featuring some data extracted from the LjGEA dataset.

As expression datasets are multidimensional, we have devised a simple, two-table-based system to accommodate the data (Fig. 4). The “metadata” table contains all metadata associated with each column, such as the age of the plant, the treatment type and/or inoculation pressure. Contents of these metadata fields is fed into Lunr.js^41^ for in-browser full-text search. The “data” table contains all the expression data of each dataset. Each row in the “data” table presents a unique gene or probe. Each row is tagged with a unique identifier in the first column, followed by three sets of columns representing the raw data: the “sample values” column, where raw expression levels are delimited with an underscore; the “sample mean” column, where the arithmetic average of raw expression levels is stored; and the “standard deviation” column, where the sample standard deviation of raw expression levels is stored. There is therefore a one-to-three relationship between the “metadata” and “data” tables, as each condition maps to three independent data columns.

### Data transformation

For easing quick visual comparison across genes with significantly different levels of absolute expression, measured by either (1) reads per kilobase of transcript (RPKM) for RNAseq datasets, or (2) arbitrary Affymetrix units for Affymetrix MicroArray datasets, we included two possibilities to transform the expression levels, by normalization or standardization. Data normalization is simply the rescaling of expression values to fit the domain [0, 1], by subtracting the log-transformed sample expression levels, *x*_*s*_, with the lowest log-transformed expression level, (log_10_ *x*)_*min*_, followed by the division of the difference between the log-transformed maximum and minimum expression levels, as defined in equation (1). In order to allow comparison for extreme values, expression values are log_10_-transformed prior to normalization.

Meanwhile, data standardization^10^ serves to rescale the expression levels on a per row basis, across conditions, to have a mean of zero and a standard deviation of one. This is performed by subtracting the sample expression levels (*x*_*s*_) by the average expression level (*μ*) across all samples, and dividing the difference with the sample standard deviation computed across all samples (*σ*), as defined in equation (2).


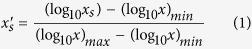



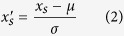


### Clustering

Depending on the size of the matrix, we implemented either *k*-means clustering (for 1-by-n or n-by-1 matrices), or hierarchical agglomerative clustering (for matrices the size of, or larger than, 2-by-2). The clustering is performed asynchronously on the server-side using SciPy^42^. As clustering is based on heuristics and therefore non-deterministic in nature, users are encouraged to export the sorted order of either, or both axes, should they want to preserve the exact clustering order.

For *k*-means clustering, the default number of starting clusters is set to the square root of the number of conditions queried, rounded up to the nearest integer. For hierarchical agglomerative clustering, the cluster cutoff is set to 0.25 of the maximum cluster distance for both axes, and is allowed to vary between 0 and 1. Complete linkage is used by default, with the option of switching to single, centroid, median, ward, or weighted methods. The default linkage metric used is Euclidean, with other options available: Braycurtis, Canberra, Chebyshev, city block (Manhattan), correlation, cosine, standard Euclidean, squared Euclidean, normalized Hamming, Jaccard, or Minkowski.

### CORNEA and CORGI: co-expression gene network visualization and co-expressed gene list retrieval

The co-expression (CORx) toolkit comprises the Co-Expression Network Analysis (CORNEA) and Co-expressed Gene Identifier (CORGI) tools. ExpAt and CORx toolkit share the same expression datasets. Co-expression gene networks in CORNEA are generated on the fly by a dedicated virtual server, which returns JSON-formatted data used for asynchronous network visualization with Sigma.js^43^ in the web browser. CORGI performs a similar function to CORNEA, but instead of generation a two-dimensional co-expression network, simply retrieves a one-dimensional slice by calling a unique gene or probe identifier, which in return generates a list of highly co-expressed entities with the gene or probe of interest.

### Generating and displaying network jobs

All CORNEA and CORGI requests are handled by a central co-expression network threaded server setup implemented using Remote Python Call (RPyC)^44^. Both client and server-side logic will check for the validity of the job request, before submitting it to the server. An entry in a MySQL table is created per job for the purpose of storing user settings and metadata of the specific network. This information is freely accessible to the user and can be exported, if the user intends to recreate the network in the future, or to reuse similar settings for network generation using alternative datasets. The submission of a valid job will trigger a redirection to a job-specific URL, which will poll the server for the job status at a set interval until completion. Once the job is completed, the user will receive an email notification if they have indicated as such prior to job submission, containing links to view their live network in the CORNEA application, and to download all data associated with their network, contained in a gzipped JSON-formatted file. The file contains all the necessary information to display a co-expression network, and within it also stores network metadata such as correlation threshold, minimum cluster size, and job runtime.

Users may also visualize networks generated by previous jobs by uploading the JSON file, gzipped or decompressed, using a drag-and-drop interface implemented in CORNEA itself. Using client-side JavaScript, the browser will unzip—if the file is gzipped—and parse the JSON file, which is handed off to SigmaJS to handle the construction of the co-expression network.

**Figure 5:**
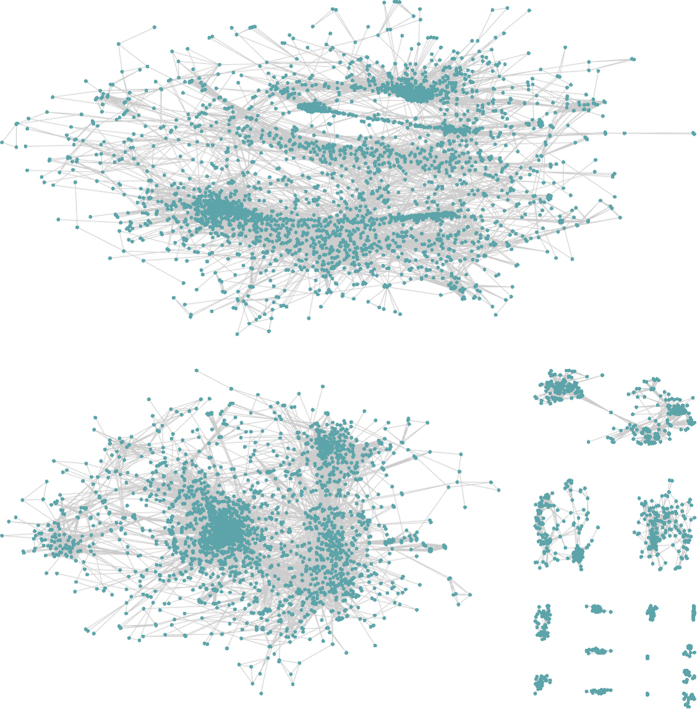
An example of a standard co-expression network generated by CORNEA, using the following parameters: LjGEA dataset with a minimum *R*^2^ value of 0.85 and a cluster size of 15 or larger. The resulting network has 7,839 nodes connected by 273,018 edges and represented in 17 distinct clusters. The network took 4 minutes and 48 seconds to generate.

We anticipate that several basic co-expression network parameters may be heavily utilized, and in order to reduce the load on the server on generating identical or highly similar networks, we have therefore generated static networks that users can utilize for preliminary exploration. An example of a static network is one that was generated from expression data from the LjGEA dataset with an *R*^2^ threshold of 0.85, and a minimum cluster size of 15. The resulting network was produced in 4 minutes and 48 seconds, with a total of 7,839 nodes—connected by 273,018 edges and found in 17 mutually exclusive clusters (Fig. 5).

As CORNEA relies heavily on client-side JavaScript on parsing and displaying the co-expression network, the use of a modern, standards-compliant browser with an optimized, efficient JavaScript engine is strongly recommended.

### Computation of co-expression relationships

Prior to pairwise calculation of correlation scores among genes or probes (collectively termed “candidates” hereon), the raw dataset is filtered in order to exclude candidates with highly similar expression pattern across conditions. For a dataset containing *N* number of candidates with a gene expression profile of c_i_, the candidate will be removed from analysis if its pattern falls below a dissimilarity threshold compared to another gene expression profile c*j* as seen in equation (3), while making exceptions for highly similar patterns with obvious peaks as defined in equation (4).


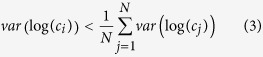



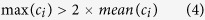


The degree of co-expression of genes is calculated as the squared Pearson’s correlation coefficient (*R*^2^) between gene and/or probe pairs across conditions. Prior to submission of a CORNEA network generation job, the user is provided with an option to subset their conditions of interest from a list of all conditions available for a given dataset.

### Node highlighting

To allow easy identification of the node(s) of interest, we implemented a highlight feature which allows the end-user to filter the displayed nodes in the network by (1) searching for a specific node, using an appropriate identifier depending on the type of dataset used, such as a gene identifier for the LjGEA dataset; or by (2) highlighting an array of nodes using a CSV file. The CSV file should contain no headers, and two columns—the first column containing the appropriate identifier for the queried dataset, and the second (optional) column containing arbitrary grouping (see supplementary, “File format for advanced node highlighting in CORNEA”). Additional columns in the CSV file will not be parsed, but can be used to store additional metadata.

### Public API

To allow other developers to benefit from the scope of our *Lotus* data, we have developed a public API using Slim framework^45^, a PHP Standard Recommendation (PSR) 7-compliant^46^ representational state transfer conformant (REST) service. All API calls are to be authenticated with a secure and cryptographically generated JWT known as an API access token. API access tokens are freely available to developers who have signed up for an account with *Lotus* Base. Due to the possibility to forge HTTP referral headers, we do not enforce domain-based restrictions on API access tokens. However, any API access token can be revoked at the liberty of developers who have created them, in the event of suspicious use by unauthorized third parties.

*Lotus* Base API uses a versioning system in order to maintain compatibility with developers using various versions of the API, to account for the possibility of major updates and changes. The *Lotus* Base API is currently at version 1, and is accessible at https://lotus.au.dk/api/v1. Complete documentation of the *Lotus* Base API v1 is available at https://lotus.au.dk/docs/api/v1.

### User accounts

Users may opt to sign up for a new account with *Lotus* Base for a more personalized experience. We have integrated several popular OAuth 2.0 identitiy providers—LinkedIn, GitHub, and Google—so that users can use alternative online services acting as identity providers to sign in, without the need to sign up manually. Existing users may also opt to integrate their *Lotus* Base user accounts with the aforementioned identity providers. *Lotus* Base adopts an ethical design principle giving users control over their own data and accounts. Private information of users is never shared with unaffiliated third parties, and their login credentials cryptographically salted and encrypted.

## Usage and Application

As a proof-of-concept use of *Lotus* Base for a typical end user, we will choose to work with *LjFls2*, the *Lotus* ortholog of *Arabidopsis FLS2 (AtFLS2*). *AtFLS2* encodes a bacterial flagellin receptor and is an important component in the induction of an evolutionarily conserved, first line defense responses in plants against pathogens^47^. The functionality of the *Lotus* ortholog, *LjFls2*, has also been previously confirmed^48^.

### Identification and BLAST search for a *Lotus* ortholog of *AtFLS2*

The amino acid sequence of *AtFLS2* (AT5G46330) was obtained from Araport^49^, and searched against the *L. japonicus* MG20 v3.0 protein database in *Lotus* BLAST. The top candidate was Lj4g3v0281040.1 with an E-value of 0 and a matching length of 1157. There were no other candidates with this degree of similarity, and a reverse BLASTp performed using the amino acid sequence of Lj4g3v0281040.1, retrieved using the SeqRet tool, against the *Arabidopsis* TAIR10 protein database revealed *AtFLS2* as the single, high-confidence match. Therefore, Lj4g3v0281040.1 is tentatively named *LjFls2* and referred to as such hereon.

### *LjFls2* is strongly expressed in *Lotus* roots

Next, we checked the expression of *LjFls2* and compared it against the closest *Lotus* homologs of a handpicked subset of genes with distinct expression patterns in plant development using ExpAt (Table 4). We selected homologs of *AtEIR1*^50^; *AtSUC2, AtCOB, AtRHD3*^51^; and members of the cellulose synthase family, *CesA* family^52^, for their root-restricted expression. We also selected members of the *SEPALATA* family for their role in flower development^53^; *AtZIFL1* and *AtZIFL2* for their upregulated expression under draught conditions^54^; and members of the alpha-galactosidase family for their role in seed development in *Arabidopsis*^55^ and tomato^56^.

**Figure 6:**
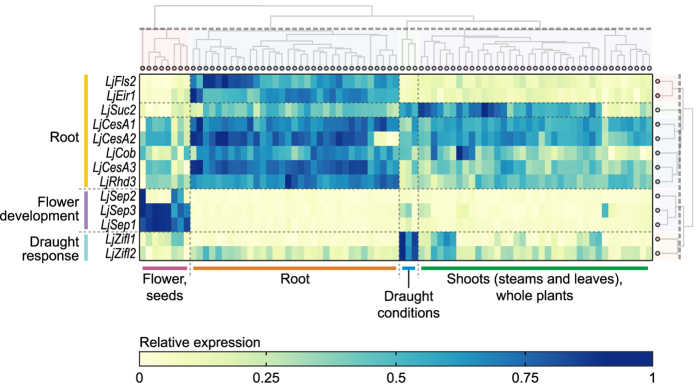
The expression heatmap generated by ExpAt for our candidate gene, *LjFls2* (Lj4g3v08201040), and other selected gene with distinct expression patterns. Expression levels, expressed as arbitrary Affymetrix units in the vertical axis, are normalized across conditions (horizontal axis). Hierarchical agglomerative clustering was performed to generate a clustered heatmap, using complete linkage over a Euclidean distance matrix, with a clustering cutoff set to 0.4.

We discovered that *LjFls2* has an expression pattern that strongly mirrors that of *LjEir1, LjSuc2, LjCob, LjRhd3*, and the *CesA* family members that show root expression in *Arabidopsis*, but not those of genes involved in other developmental stages and/or organs (Fig. 6). Hierarchical clustering was performed in ExpAt, using a Euclidean distance matrix over complete linkage based on squared Pearson’s correlation values (*R*^2^). This revealed distinct clusters of genes and conditions, with genes clustering into groups demarcated by developmental stage and organ in *Arabidopsis*, and conditions clustering into groups defined by organs and treatment conditions (Fig. 6).

### *LjFls2* is located in the same co-expression cluster as genes with root-based expression

**Figure 7:**
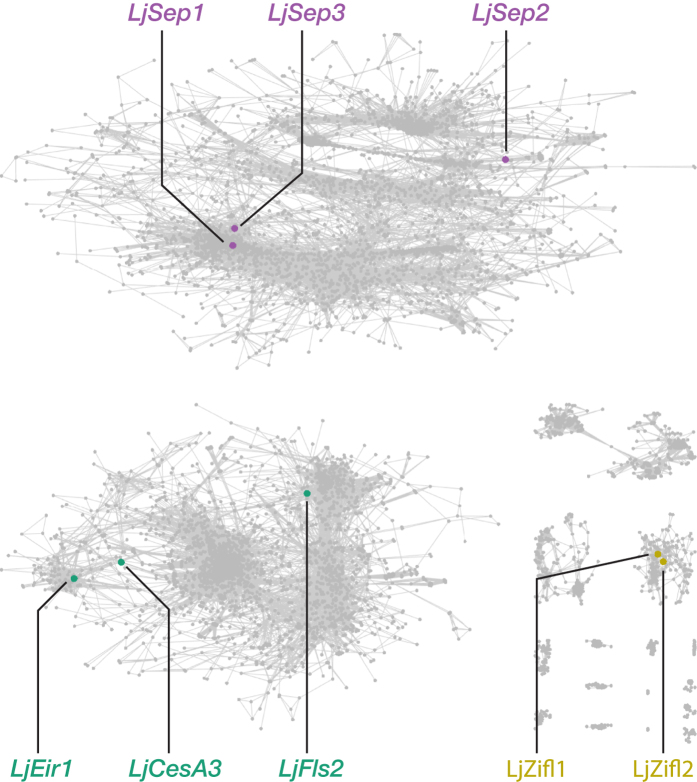
The highlighted nodes of LjFls2 and other selected genes (see Table 4) in a standard co-expressed genes network map generated from the LjGEA dataset, using an R2 threshold of 0.85 and a minimum cluster size of 25. Some root-based genes—*LjCob, LjRhd3, LjSuc2, LjCesA1*, and *LjCesA2*—were not found in the network, due to their expression patterns not meeting the minimum threshold on the squared Pearson’s correlation score (*R*^2^). Abbreviations: *CesA*, cellulose synthase family; *Cob*, COBRA-like extracellular glycosyl-phosphatidyl inositol-anchored protein family; *Fls2*, flagellin-sensing 2; *Rhd3*, root hair defective 3; *Suc2*, sucrose-proton symporter 2.

In order to visualize the co-expression network around *LjFls2*, we loaded the standard network generated from the LjGEA dataset in CORNEA, and highlighted network nodes using the gene list in Table 4 (Fig. 7; see supplementary “Node highlighting in CORNEA with selected genes”). Even when genes strongly expressed in the roots do not show highly correlated expression pattern (*R*^2^ ≤ 0.85) with *LjFls2*, they are still found in the same mega cluster, suggesting overall similarities in expression patterns. More importantly, flower development genes *SEPALATA* are found in another distinct mega cluster, and so are those involved in draught responses, *LjZifl1* and *LjZifl2*.

Taken together, this suggests that both ExpAt and CORNEA are reliable tools in not only differentiating, but correctly clustering, distinct gene expression patterns in *Lotus*. Moreover, both tools complement each other by providing a different perspective on the relationship of the expression patterns between candidate genes—ExpAt allows inference of relationship(s) among user-defined candidates, while CORNEA provides spatial information on how user-defined candidates fit into the overall expression network generated from a dataset.

### Genes that are strongly co-expressed with *LjFls2* have been functionally validated

**Figure 8:**
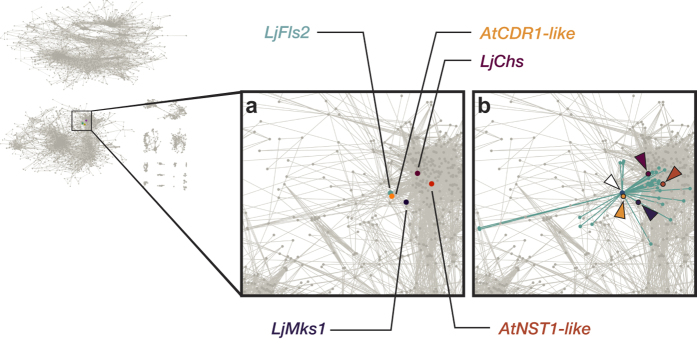
The location of four strongly co-expressed genes, relative to *LjFls2*, in the standard gene co-expression network generated from the LjGEA dataset. Insets depict (**a**) the highlighted nodes of the three genes respectively, and (**b**) the immediate network around *LjFls2*, which contains co-expressed genes that meet the minimum threshold of *R*^2^ ≥ 0.85. Among them are *AtCDR1-like* (orange), *AtNST1-like* (red), *MtCHS1-like* (maroon), and *AtMKS1-like* (black). The white-filled triangle indicates the root candidate gene, *LjFls2*, which is partially occluded by an overlying node in (**b**). Abbreviations: *CDR1*, constitutive disease resistance 1; *Chs*, chalcone synthase; *Fls2*, flagellin-sensing 2; *MKS1*, mitogen-activated protein kinase substrate 1; *NST1*, no apical meristem (NAC) secondary wall thickening promoting factor 1.

CORGI was used to generate a list of the top 25 highly co-expressed genes of *LjFls2* (Table 5), and putative *Lotus* orthologs of four candidates whose expression patterns have been verified by published literature to be correlated with, or induced by, flagellin exposure—*AtCDR1-like, AtNST1-like, MtCHS1-like*, and *AtMKS1-like*. These genes were found not only in the same co-expression megacluster, but also directly connected to LjFLS2 in the network (Fig. 8).

Lj6g3v1880370 (1^st^, *R*^2^ = 0.933) is highly similar to a gene encoding for an aspartyl protease-like protein in *Arabidopsis*. A gene encoding an apoplastic aspartyl protease, *AtCDR1*, is found to play an important role in conferring salicylic acid-dependent resistance against *Pseudomonas syringae* in *Arabidopsis*^57^. Although the role of proteases in defense responses are yet to be clearly elucidated, it is hypothesized that they either aid in the processing of *R* proteins, or through enzymatic action generate ligands that are recognized by *R* proteins^58^,^59^,^60^.

Lj4g3v2603590 (2^nd^, *R*^2^ = 0.911) encodes a *NST1-like* protein, a member of a family of genes involved in the regulation of secondary cell wall thickening in *Arabidopsis*^61^ due to its role in lignin biosynthesis^62^. Lignification of plant cell walls may be induced by mechanical, environmental and disease stresses^63^,^64^, and treatment with bacterial flagellin has shown to induce lignin biosynthesis in plants^65^,^66^,^67^.

Lj4g3v2574990 (4^th^, *R*^2^ = 0.899) is a chalcone synthase (CHS) found in both alfalfa (*Medicago truncatula*) and Mexican lime (*Citrus aurantifolia* L.), and its expression is upregulated upon exposure to flagellin of their respective pathogens, *Aphanomyces euteiches*^68^ and *Candidatus* Phytoplasma aurantifolia^69^.

Lj2g3v1155180 (5^th^, *R*^2^ = 0.897) is the closest homolog of the *Arabidopsis MKS1* (At3G18690), which encodes a protein that is substrate of AtMPK4^70^, a kinase involved in the regulation of defense responses in plants^71^. More poignantly, AtMPK4 is activated by exposure to flagellin purified from *P. syringae*, an adapted pathogen of *Arabidopsis*, and results in phosphorylation of AtMKS1.

### Multiple *LORE1* lines with exonic insertions in *LjFls2*

Next, we retrieved *LORE1* mutant lines that contain exonic insertions in the *LjFls2* gene using the TREX tool. Out of the 40 *LORE1* lines that contain insertions in *LjFls2*, 31 are exonic, of which 29 originate from the Danish collection and are therefore orderable through *Lotus* Base (Table 6). These 29 lines can be propagated (as F0 plants) and allowed to self-fertilize in order to generate F1 homozygous mutant lines, whose progenies (F2) will be useful for further phenotyping studies, if desired.

## Discussion

In this paper, we introduced *Lotus* Base, an integrated information portal for genomic and expression data for the model legume *L. japonicus*. With the utilization of modern browser technology and cryptographically secure information transmission, *Lotus* Base poises itself to be at the forefront of accessibility, security, privacy and usability of large-scale scientific data without sacrificing usability. The lack of a central database for *Lotus* resources has been a strong driving force behind the creation of *Lotus* Base. This places *Lotus japonicus* on par with other popular model plants, such as *A. thaliana, G. max*, and *M. truncatula*, all of which have dedicated online platforms that serve integrated data, namely Araport^49^, the *Arabidopsis* Information Resource^72^, SoyBase^73^ and the *Medicago truncatula* Genome Database^74^.

*Lotus* Base distinguishes itself from other cross-species integration platform such as Legume Information System (LIS)^75^,^76^, PlantGDB^77^, and Phytozome^78^, by offering comprehensive species-specific data. In addition, *Lotus* genomic data is available on LIS^75^,^76^, PlantGDB^77^, Phytozome^78^, and through the Kazusa DNA Research Institute website^9^; and *Lotus* expression data on LjGEA^10^. However, there are hitherto neither *LORE1* mutant population nor *Lotus* expression data integrated with the sequenced and annotated genome of *Lotus*. Yet, similar to the motivation behind Araport^49^ and LIS, *Lotus* Base is designed in response to a fragmented landscape of *Lotus* data available across various platforms, by bridging data sourced from various studies. The integration of various resources, such as the search and order system of 120,000+ *LORE1* lines, the assimilation and deep linking of publicly available expression datasets, makes *Lotus* Base a convenient and feature-rich one-stop repository for *Lotus* resources. While other legume resources offer similar datasets, many features are non-standards compliant, rely on dated web technologies, lack user friendliness, or do not offer integrated data for easy data mining (Table 7). Moreover, the web-based implementation of *Lotus* Base aims to improve data availability and exchange among the *Lotus* research community, unimpeded by computer hardware and operation systems, or technological know-how of the end user.

The modular construction and open-source model of *Lotus* Base ensure continuity and encourage expansion and inclusion of additional dataset with relative ease in the future. In addition, the public API of *Lotus* Base aims to benefit a larger community by making *Lotus* data available to developers who are deploying applications that pull integrated data from our databases.

The introduction of *Lotus* BLAST allows deep integration of *Lotus* BLAST databases with other toolkits specifically designed to tackle data visualization and analysis. The implementation of various toolkits such as ExpAt, CORNEA and CORGI can be extrapolated to datasets unrelated to *Lotus*, or even scientific research in general. We demonstrated that ExpAt offers users a powerful way of visualizing co-expression relationships on a subset of user-defined candidates by leveraging on *k*-means or hierarchical clustering, while CORNEA presents users a two-dimensional, spatial chart of co-expression relationships among all genes from selected datasets. The use of data-driven documents in these toolkits reveal their prowess in the ability to visualize large volumes of data with ease, by combining the computational power of server-side technologies and the efficiency of client-side JavaScript interpreters. Many features on *Lotus* Base can therefore be adapted by the community as novel ways to represent, investigate, analyze, and visualize biological data. We believe that *Lotus* Base will not only make comprehensive *Lotus* data accessible to researchers easily, but also empower them to perform computationally intensive and complex analysis and visualization without the need for extensive technological skills. Taken in all, *Lotus* Base will benefit the legume research community and beyond, by providing a framework for a coherent scientific workflow and powerful tools for raw data interpretation.

## Additional Information

**How to cite this article**: Mun, T. *et al.* *Lotus* Base: An integrated information portal for the model legume *Lotus japonicus. Sci. Rep.* **6**, 39447; doi: 10.1038/srep39447 (2016).

**Publisher's note:** Springer Nature remains neutral with regard to jurisdictional claims in published maps and institutional affiliations.

## Supplementary Material

Supplementary Information

## Figures and Tables

**Table 1:** List of datasets available through *Lotus* Base.

Dataset	Toolkits					
	*Lotus* BLAST	*LORE1* search	Genome browser	CORx toolkit	ExpAt	Gene page
**Genome**						
*L. japonicus* MG20 v2.5 genome	+	+	+	−	−	−
*L. japonicus* MG20 v3.0 genome	+	+	+	−	−	+
*L. japonicus* MG20 v3.0 cDNA	+	−	−	−	−	+
*L. japonicus* MG20 v3.0 CDS	+	–	−	−	−	+
**Transcriptome**						
*Lotus* responses to draught stress^33^	−	−	−	+	+	+
*Lotus* responses during arbuscular mycorrhizal symbiosis^34^	−	−	−	+	+	+
Responses in wildtype and symbiotic mutants of *Lotus* during legume-rhizobium symbiosis^35^	−	−	−	+	+	+
Salt acclimatization in *Lotus japonicus* Gifu^36^	−	−	−	+	+	+
Salt acclimatization among *Lotus* ecotypes^37^	−	−	−	+	+	+
Early *Lotus* root responses to germinating spore extract^5^	−	−	−	−^a^	+	+
**Predicted proteins**						
*L. japonicus* proteins v2.5	−	+	+	−	−	−
*L. japonicus* proteins v3.0	+	+	+	+	+	+
***LORE1*** **resource**						
Danish collection: DK01–03, 05, 07–16^8^	−	+	+	−	−	+
Japanese collection: JPA, JPL, and JPP^8^	−	+	+	−	−	+

Datasets without any references represent original datasets generated in this work.

^a^Excluded from co-expression analysis due to low number of treatments/conditions available (3 only).

**Table 2:** A non-exhaustive overview of currently available mutageneis populations for model plants.

Organism	Agent	Type	Method	Classification	Population	Reference
*A. thaliana*	T-DNA	Insertional	Agrobacterium transfection	Transgenic	48,830	79
*A. thaliana*	Ac/Ds	Insertional	Transposon	Transgenic	559	80
*A. thaliana*	*Tto1*	Insertional	Retrotransposon	Transgenic	255*	81
*A. thaliana*	*Tnt1*	Insertional	Retrotransposon	Transgenic	*ca.* 400*	82
*L. japonicus*	*LORE1*	Insertional	Retrotransposon	Non-transgenic	121,531	8
*L. japonicus*	*Tnt1*	Insertional	Retrotransposon	Transgenic	51*	83
*M. truncatula*	*Tnt1*	Insertional	Retrotransposon	Transgenic	*ca.* 12,000	84
*M. truncatula*	*Tnt1*	Insertional	Retrotransposon	Transgenic	2*	85
*Lettuce*	*Tnt1*	Insertional	Retrotransposon	Transgenic	10*	86
*Rice*	*Tos17*	Insertional	Retrotransposon	Non-transgenic	47,196	87
*Soybean*	*Tnt1*	Insertional	Retrotransposon	Transgenic	27*	88
*A. thaliana*	EMS	Allelic SNP	TILLING	Non-transgenic	6,764	89, 90
*L. japonicus*	EMS	Allelic SNP	TILLING	Non-transgenic	8,556	91
*M. truncatula*	EMS	Allelic SNP	TILLING	Non-transgenic	3,162	92
*Rice*	EMS, NaN_3_, NMU	Allelic SNP	TILLING	Non-transgenic	5,120	93, 94
*Soybean*	EMS	Allelic SNP	TILLING	Non-transgenic	116	95
*Tomato*	EMS, NMU	Allelic SNP	TILLING	Non-transgenic	5,508	96
*Wheat*	EMS	Allelic SNP	TILLING	Non-transgenic	1,536	97

Mutagenesis methods that have so far only established starter lines without generating a large-scale mutagenis population are marked with an asterisk (*). Abbreviations: EMS, ethyl methanesulfonate; NMU, *N*-nitroso-*N*-methylurea, SNP, single nucleotide polymorphism; TILLING, Targeting Induced Local Lesions in Genomes.

**Table 3:** A table describing all *LORE1*-associated data available through *Lotus* Base.

Field	Description	Example value
PlantID	A seven-digit mutant plant identifier, in the format of 3xxxxxxx.	30000001
Batch	The sources of the *LORE1* line, indicating its Danish (DKn) or Japanese origins (JPx).	DK01
ColCoord	Column coordinate of the plant for FSTpoolit. This value can be used to resolve lines with identical *LORE1* inserts.	C_1
RowCoord	Row coordinate of the plant for FSTpoolit. This value can be used to resolve lines with identical *LORE1* inserts.	R_1
Chromosome	The chromosome which the *LORE1* insert is mapped to. This value may differ among genome assembly versions.	chr0
Position	Position of the *LORE1* insert.	146086605
CoordList	Pool coordinate details of all lines containing a particular *LORE1* insert. Used for resolving lines with identical inserts.*Note: In the example value, mutant plants containing the same insertion are observed to originate from two coordinates, C_1#R_1 and C_49#R_43.*	C_1#C_49#R_1#R_43
CoordCount	Absolute counts of the number of reads associated with each pool coordinate.*Note: In the example value, C_1 and R_1 have the highest column and row counts (89 and 215 respectively), therefore the mutant line with coordinates C_1#R_1 is likely the actual mutant line containing this particular insert.*	89#27#215#9
TotalCoverage	Sequencing coverage at the *LORE1* insert.	340
FwPrimer	Forward primer designed using Primer3, based on the ±1000 flanking sequence.	TGCCAGCACCTGCAAATGAGAATCA
RevPrimer	Reverse primer designed using Primer3, based on the ±1000 flanking sequence.	TGTCCAGGTCTTGCTGCCAAATCA
PCRInsPos	Size of PCR product if there is a positively identified *LORE1* insert.	516
PCRWT	Size of PCR product if there is no *LORE1* insert.	507
InsFlank	The ±1000 bp flanking sequence (2 kb int total) of the *LORE1* insert.	TGTTTTCACCTTATATCTCT
Ordering*	A Boolean value indicating if the line is orderable or not.	1
Version	*Lotus* genome assembly version against which the *LORE1* line is mapped.	3.0
Salt*	A unique 32-character hexadecimal identifier of a *LORE1* insert.	7cd0a4c8e9f10c4096d1782702267c59
IDKey*	An auto-incremental, unique entry identifier for indexing purposes.	131071

Rows that are not made available through data export are marked with an asterisk (*).

**Table 4:** List of handpicked genes for visualization in ExpAt and CORNEA, based on their expression in developmental stages and organs in *Arabidopsis*.

*Lotus* organ	*Arabidopsis* gene		Putative *Lotus*orthologs	
	ID	Name	ID	Name
Root	AT5G46330	*AtFLS2*	Lj4g3v0281040	*LjFls2*
Root	AT5G57090	*AtEIR1*	Lj4g3v2139970	*LjEir1*
Root	AT1G22710	*AtSUC2*	Lj2g3v0205600	*LjSuc2*
Root	AT5G60920	*AtCOB*	Lj1g3v0414750	*LjCob*
Root	AT4G32410	*AtCESA1*	Lj0g3v0249089	*LjCesA1*
Root	AT4G39350	*AtCESA2*	Lj4g3v2775550	*LjCesA2*
Root	AT5G05170	*AtESA3*	Lj0g3v0245539	*LjCesA3*
Root	AT3G13870	*AtRHD3*	Lj3g3v2693010	*LjRhd3*
Flower development	AT1G24260	*AtSEP3*	Lj4g3v2573630	*LjSep3*
Flower development	AT5G15800	*AtSEP1*	Lj2g3v1105370	*LjSep1*
Flower development	AT3G02310	*AtSEP2*	Lj4g3v1736080	*LjSep2*
Draught tolerance	AT5G13750	*AtZIFL1*	Lj1g3v2975920	*LjZifl1*
Draught tolerance	AT3G43790	*AtZIFL2*	Lj6g3v1052420	*LjZifl2*

Genes were separated into three groups: root, flower development and draught tolerance. *Lotus* orthologs are discovered by performing a BLASTp search of the corresponding *Arabidopsis* genes against *L. japonicus* MG20 proteins v3.0 database, and by selecting the candidate of the highest confidence selected. *Lotus* orthologs inherit the name of their *Arabidopsis* counterparts, with the standard gene nomenclature used for *Lotus*. Abbreviations: *CesA*, cellulose synthase family; *Cob*, COBRA-like extracellular glycosyl-phosphatidyl inositol-anchored protein family; *Eir1*, ethylene-insensitive root 1; *Fls2*, flagellin-sensing 2; *Rhd3*, root hair defective 3; *Sep*, SEPALATA family; *Suc2*, sucrose-proton symporter 2; *Zifl*, zinc-finger-like protein family.

**Table 5:** The top 25 highly co-expressed genes of *LjFls2*, generated by CORGI.

Gene ID	Name/Description	*R*^2^
Lj6g3v1880370	PREDICTED: basic 7S globulin-like [*Glycine max*] gi|356557887|ref|XP_003547241.1|	0.93336
Lj4g3v2603590	PREDICTED: stress response protein NST1-like [*Cicer arietinum*] gi|502140841|ref|XP_004504356.1|	0.91121
Lj0g3v0320039	PREDICTED: probable receptor-like protein kinase At5g20050-like [*Glycine max*] gi|356563053|ref|XP_003549780.1|	0.90673
Lj4g3v2574990	chalcone synthase CHS4 [*Glycine max*] gi|34148079|gb|AAQ62588.1|	0.89932
Lj2g3v1155180	Protein MKS1 [*Medicago truncatula*] gi|357444747|ref|XP_003592651.1|	0.89682
Lj0g3v0173689	PREDICTED: wall-associated receptor kinase 5-like [*Glycine max*] gi|356551203|ref|XP_003543967.1|	0.89649
Lj3g3v0602640	n.a.	0.88933
Lj3g3v0602630	phenylalanine ammonia-lyase [*Lotus japonicus*] gi|118142392|dbj|BAF36971.1|	0.88933
Lj3g3v0602620	phenylalanine ammonia-lyase [*Lotus japonicus*] gi|118142392|dbj|BAF36971.1|	0.88933
Lj1g3v4590760	phenylalanine ammonia-lyase [*Lotus japonicus*] gi|118142392|dbj|BAF36971.1|	0.88901
Lj2g3v1369250	PREDICTED: zinc finger protein 5-like [*Cicer arietinum*] gi|502141274|ref|XP_004504507.1|	0.88272
Lj6g3v1418060	PREDICTED: zinc finger protein 5-like [*Cicer arietinum*] gi|502141274|ref|XP_004504507.1|	0.88272
Lj0g3v0245779	n.a.	0.87638
Lj0g3v0245769	n.a.	0.87638
Lj0g3v0305019	Uncharacterized protein TCM_040942 [*Theobroma cacao*] gi|508785660|gb|EOY32916.1|	0.87633
Lj4g3v2578250	Rhg4-like receptor kinase II [*Glycine max*] gi|90655934|gb|ABD96566.1|	0.87422
Lj1g3v4590840	phenylalanine ammonia-lyase [*Lotus japonicus*] gi|118142384|dbj|BAF36967.1|	0.87387
Lj3g3v1421800	PREDICTED: U-box domain-containing protein 16-like [*Cicer arietinum*] gi|502146392|ref|XP_004506434.1|	0.87052
Lj3g3v1421810	PREDICTED: U-box domain-containing protein 16-like [*Cicer arietinum*] gi|502146392|ref|XP_004506434.1|	0.87052
Lj0g3v0343089	n.a.	0.86410
Lj6g3v0958320	Uncharacterized protein TCM_040942 [*Theobroma cacao*] gi|508785660|gb|EOY32916.1|	0.86281
Lj2g3v2051190	PREDICTED: 1-aminocyclopropane-1-carboxylate synthase-like [*Glycine max*] gi|356539620|ref|XP_003538294.1|	0.86203
Lj1g3v3716560	GntR family transcriptional regulator [*Cupriavidus basilensis*] gi|493149530|ref|WP_006161625.1|	0.86103
Lj1g3v3716780	n.a.	0.86103
Lj1g3v0129110	n.a.	0.86103

The candidates were pulled from a one-dimensional slice across the co-expression matrix generated by CORNEA, ranked by the squared Pearson’s correlation coefficient (*R*^2^) in descending order. CORGI returns 25 rows by default, but may be configured to return up to 100 candidates.

**Table 6:** All *LORE1* lines containing a *LORE1* insert in the *LjFls2* gene, found across 16 batches of *LORE1* populations.

*LORE1* ID	Batch	Chromosome	Position	Orientation	Insertion type
30003492	DK01	chr4	3287060	F	Exonic
30012416	DK03	chr4	3286059	R	Exonic
30030341	DK05	chr4	3288262	F	Intronic
30030425	DK05	chr4	3286258	F	Exonic
30033827	DK05	chr4	3286209	F	Exonic
30034607	DK05	chr4	3287742	F	Exonic
30035947	DK05	chr4	3287150	F	Exonic
30056942	DK07	chr4	3287104	F	Exonic
30057743	DK07	chr4	3284745	R	Exonic
30057897	DK07	chr4	3285808	F	Exonic
30060694	DK08	chr4	3285199	F	Exonic
30003492	DK01	chr4	3287060	F	Exonic
30012416	DK03	chr4	3286059	R	Exonic
30061005	DK08	chr4	3285639	R	Exonic
30070461	DK09	chr4	3288029	R	Intronic
30071709	DK09	chr4	3288029	R	Intronic
30072232	DK09	chr4	3286020	R	Exonic
30072618	DK09	chr4	3287504	R	Exonic
30074013	DK09	chr4	3288029	R	Intronic
30075601	DK09	chr4	3286721	F	Exonic
30080413	DK10	chr4	3285894	F	Exonic
30083670	DK10	chr4	3285699	R	Exonic
30084653	DK10	chr4	3287937	R	Intronic
30088736	DK11	chr4	3284973	R	Exonic
30092255	DK11	chr4	3285297	R	Exonic
30095950	DK12	chr4	3285585	F	Exonic
30100097	DK12	chr4	3288274	R	Intronic
30100269	DK12	chr4	3286310	F	Exonic
30108970	DK13	chr4	3286450	R	Exonic
30109089	DK13	chr4	3287032	R	Exonic
30109124	DK13	chr4	3288292	F	Intronic
30109659	DK13	chr4	3284933	R	Exonic
30115606	DK14	chr4	3284648	R	Exonic
30117374	DK14	chr4	3285311	F	Exonic
30119789	DK14	chr4	3286700	R	Exonic
30119801	DK14	chr4	3286700	R	Exonic
30124389	DK15	chr4	3286500	F	Exonic
30138429	DK16	chr4	3285596	F	Exonic
A04405	JPA	chr4	3287360	R	Exonic
L0530	JPL	chr4	3288070	R	Intronic
L4758	JPL	chr4	3286032	F	Exonic
P1585	JPP	chr4	3288189	R	Intronic

Abbreviations: chr, chromosome; F, forward; R, reverse.

**Table 7:** Comparison of features available on extant legume resources and *Lotus* Base.

	Legume Information System^1^ ^75^	*Lotus japonicus*^2^ or *Medicago truncatula*^3^ Gene Expression Atlas (Lj/MtGEA)^10^,^98^,^99^	Kazusa DNA Research Institute^4^ ^9^	*Medicago truncatula* Genome Project^5^ ^74^	SoyBase^6^ ^73^	*Lotus* Base^7^ (this work)
**Species**	21	1; *L. japonicus* or *M. truncatula*	1; *L. japonicus*	1; *M. truncatula*	1; *G. max*	1; *L. japonicus*
**Genome browser**	Yes; GBrowse^100^ and JBrowse^12^	Yes; for MtGEA only (available as external link)	Yes; GBrowse	Yes; JBrowse	Yes; GBrowse	Yes; JBrowse
**Genetic map**	No	No	Yes	Yes	Yes	No
**Gene ontology**	Yes	No	Yes	Yes	Yes	No
**Data mining**	Partial (only 3 species supported)	No	Yes	Yes	Yes	Yes
- Implementation	Intermine^101^	No	Custom-designed solution	Intermine	Intermine	Custom-designed solution
**Large-scale mutagenesis population & data**	No	No	No	No	No	Yes; 121,531 *LORE1* lines containing 629631 unique insertions
- Predicted gene model overlay	No	No	No	No	No	Yes
- Ordering and dispatching	No	No	No	No	No	Yes
**BLAST**	Yes; NCBI BLAST^39^	Yes; NCBI BLAST	Yes; NCBI BLAST	Yes; NCBI BLAST	Yes; NCBI BLAST	Yes; SequenceServer^38^ powered by NCBI BLAST
- Programs						
- - blastn	Yes	Yes	Yes	Yes	Yes	Yes
- - blastx	Yes	No	Yes	Yes	Yes	Yes
- - tblastn	Yes	No	Yes	Yes	Yes	Yes
- - tblastx	No	No	Yes	Yes	Yes	Yes
- - blastp	Yes	No	Yes	Yes	Yes	Yes
- Datasets						
- - Genome	Yes	No	Yes	Yes	Yes	Yes
- - cDNA/mRNA	No	No	Yes	Yes	Yes	Yes
- - CDS	Yes	No	Yes	Yes	Yes	Yes
- - Proteins	No	No	Yes	Yes	Yes	Yes
- - Misc	–	Microarray chip target, sequences, and probes	–	Unspliced transcripts and BAC ends	–	LjGEA microarray chip probes
**Gene expression**	No	Yes	No	No	Yes	Yes
- Data transformation	No	Yes; normalization	No	No	Yes; normalization	Yes; normalization or standardization
- Data export	No	Yes; values are available as replica readouts or arithmetic means	No	No	Yes; only single values available in CSV format	Yes; values are available as replica readouts, arithmetic means, and pre-computed standard deviations
- Visualization	No	Yes; reliance on Adobe Flash, requires multiple windows to be opened	No	No	Yes; rudimentary and tabular based	Yes; using web-based data-driven approach, highly customizable
- Analysis	No	No	No	No	Yes; hierarchical clustering	Yes; asynchronous clustering—*k*-means or hierarchical—dependent on matrix size
- Co-expression analysis	No	Yes; single dimensional co-expression relationships	No	No	No	Yes; single and two-dimensional co-expression relationships, spatial network construction, and data-driven presentation
**Public API**	No	No	No	No	No	Yes
**User documentation & help**	Yes	Yes	No	Yes	Yes	Yes

Abbreviations: BLAST, basic local alignment search tool; CDS, coding sequence; GEA: gene expression atlas.

^1^http://legumeinfo.org.

^2^http://ljgea.noble.org/v2/.

^3^http://mtgea.noble.org/v3/.

^4^http://www.kazusa.or.jp/lotus/.

^5^http://medicago.jcvi.org/MTGD/.

^6^http://soybase.org.

^7^https://lotus.au.dk.
