# Supplementary material for: Lotus Base: An integrated information portal for the model legume Lotus japonicus
Source: Sci Rep. 2016 Dec 23;6:39447. doi: 10.1038/srep39447 (PMC5180183; doi:10.1038/srep39447)
Supplement: Supplementary Information [file srep39447-s1.doc]

# **Title:**

*Lotus* Base: An integrated information portal for the model legume *Lotus japonicus*

# **Authors:**

Terry Mun^1^*, Asger Bachmann^1,2^, Vikas Gupta^1,2^, Jens Stougaard^1^, and Stig U. Andersen^1^*

# **Author affiliations:**

^1^ Centre for Carbohydrate Recognition and Signalling, Aarhus University

^2^ Bioinformatics Research Centre, Aarhus University

# **Authors for correspondence:**

Terry Mun (email: [terry@mbg.au.dk](mailto:terry@mbg.au.dk)) and Stig Uggerhøj Andersen (sua@mbg.au.dk)

## SUPPLEMENTARY INFORMATION

### **File format for advanced node highlighting in CORNEA**

A valid example of an uploaded CSV file is as follow:

| GeneA, group1  GeneB, group1  GeneC, group1  GeneD, "another group"  GeneE, "another group"  GeneF, 3  GeneG, 3 |
| --- |

This will instruct CORNEA to highlight GeneA, GeneB and GeneC with a single color, as they belong to a group known as “group1”, while GeneD and E with another color, in the group “another group”. Both strings and numbers can be used to denote grouping.

By default, groups are highlighted using ColorBrewer’s Dark2 palette. A user-defined color palette may be used, as long as a valid hexadecimal or RGB color is used as a string in the grouping column:

| GeneA, #45B29D  GeneB, #45B29D  GeneC, #45B29D  GeneD, "rgb(223,90,73)"  GeneE, "rgb(223,90,73)"  GeneF, "rgb(223,90,73)"  GeneG, "rgb(223,90,73)" |
| --- |

If certain rows are missing the second column, a random 8-character hexadecimal string will be assigned to said rows as a group identifier. All rows without a group specified will be assigned to the same group. Therefore, the following input:

| GeneA, 1  GeneB, group1  GeneC, group1  GeneD  GeneE  GeneF  GeneG |
| --- |

…will be converted into the following:

| GeneA, 1  GeneB, group1  GeneC, group1  GeneD, 7937b73e  GeneE, 7937b73e  GeneF, 7937b73e  GeneG, 7937b73e |
| --- |

### **Node highlighting in CORNEA with selected genes**

The following list, constructed from candidate genes of distinct expression patterns, is used for node highlighting in CORNEA.

| Lj4g3v0281040, Root  Lj4g3v2139970, Root  Lj2g3v0205600, Root  Lj1g3v0414750, Root  Lj0g3v0249089, Root  Lj4g3v2775550, Root  Lj0g3v0245539, Root  Lj3g3v2693010, Root  Lj4g3v2573630, Flower  Lj2g3v1105370, Flower  Lj4g3v1736080, Flower  Lj1g3v2975920, "Draught tolerance"  Lj6g3v1052420, "Draught tolerance" |
| --- |
